# Emotional intelligence and self-esteem: Personal competencies necessary for physicians

**DOI:** 10.3389/fmed.2022.965417

**Published:** 2022-07-27

**Authors:** María del Mar Molero Jurado, María del Carmen Pérez-Fuentes, África Martos Martínez, José Jesús Gázquez Linares

**Affiliations:** ^1^Department of Psychology, Faculty of Psychology, University of Almería, Almería, Spain; ^2^Department of Psychology, Universidad Politécnica y Artística del Paraguay, Asunción, Paraguay; ^3^Department of Psychology, Universidad Autónoma de Chile, Providencia, Chile

**Keywords:** emotional intelligence, self-esteem, physicians, personal competencies, contract

## Abstract

**Introduction:**

Even though emotional intelligence and self-esteem are truly important variables in healthcare, the fact is that there are few studies on these constructs in Spanish physicians. The objective was to analyze the relationship between self-esteem and emotional intelligence in Spanish physicians, and their relationship with sociodemographic and employment variables.

**Methods:**

The study included a sample of 180 physicians with a mean age of 34.61, 76.7% of whom were women. The physician completed the Rosenberg self-esteem scale, the Brief Emotional Intelligence Inventory for Senior Citizens and a questionnaire on sociodemographic and work characteristics through a CAWI (Computer Assisted Web Interviewing).

**Results:**

The results showed that the interpersonal factor of emotional intelligence diminished with age and number of children. Furthermore, women scored significantly higher in interpersonal emotional intelligence and men in adaptability. Physicians with stable contracts (46.1%) scored higher in all the emotional intelligence variables, with almost a small effect size. In the relationship between self-esteem and emotional intelligence, it was found that global self-esteem of physicians was positively related to the Stress Management, Adaptability and Mood dimensions. Physicians with high self-esteem (72.8%) showed better Stress Management and Mood than those with low self-esteem (6.1%), while in the Adaptability factor, physicians with high global self-esteem scored higher than those with medium levels (21.1%).

**Conclusion:**

The need for a stable employment strategy was demonstrated, since this has repercussions on personal competencies of physicians as well as training in developing those consequences, since they improve physicians' quality of care and leadership, especially women with temporary contracts or with medium or low self-esteem.

## Introduction

Any job requiring contact with others requires a certain capacity for managing emotions, and in the specific case of medical attention, this is especially important due to the high emotional intensity of their profession (1–3). So much the more so considering that this variable is one of the most powerful predictors of life and work outcomes (4–8). Therefore, emotional intelligence is an important personal competency in this group, and better understanding of it is necessary for two reasons. The first has to do with improving employee training for their emotional competency. The second is connected to the need to include competencies related to learning and managing the emotions of experiencing disease, the attention process and patient, colleague and family relations in the educational program of future physicians (1, 9, 10).

Similarly, keeping in mind the healthcare environment, self-esteem is another very important factor in stressful settings, as it is related to confident judgement and decision-making. Furthermore, both variables (emotional intelligence and self-esteem) have demonstrated their importance in professional leadership (11, 12), which is especially relevant in care culture which is hierarchical and competitive, even though collaborative teams and cooperation for excellence are sought (13). However, medical training concentrates almost completely on technical skills, obviating these personal factors which impact fully on the results of their labor and personal adjustment (14–16).

Therefore, it is of interest to determine the factors related to self-esteem and emotional intelligence levels of physicians. Although these variables have been analyzed in other healthcare groups, such as nursing or in healthcare science students (17–19), studies in the medical profession are few, which could be related to the social perception of physicians as emotionally disconnected from others and from themselves, as invulnerable agents (20). Therefore, this study examines the factors linked to these variables and how these psychological elements are related in a sample of Spanish physicians.

According to Bar-On (21), emotional intelligence refers to the set of skills, aptitudes and competencies that influence the capacity for success in the face of environmental pressures and demands. Emotionally intelligent individuals regulate their emotions to adapt to the situation flexibly and in a manner coherent with their goals, maximizing their survival (22). Based on this definition, the EQ questionnaire is used to evaluate emotional intelligence. This questionnaire is called a mixed measure because it combines socioemotional traits, skills and competencies, whereas others evaluate emotional intelligence only as a trait or as an ability to understand emotions and how they function. In this sense, mixed measures are especially appropriate in a job context (23) such as the medical profession which is the framework of this study. The emotional intelligence model factors are Adaptability (flexibility in confronting new situations), Stress Management (tolerance and control of negative emotions), Interpersonal (related to understanding and management of the emotions of others), Intrapersonal (knowledge and control of one's own emotions) and Mood (related to optimism and happiness) (21, 24, 25).

In the scope of medical attention, those with high EI are more effective than those with low levels for fighting against the frequently stressful situations. So this variable has become established as an important predictor of performance and professional satisfaction (26, 27). Its outstanding protective role against burnout during residency and afterwards has also been described (28–30). Studies in samples of healthcare professionals indicate the need to evaluate the role of age (31) and gender in emotional intelligence (32), especially in developing programs directed at differential characteristics (2, 33). However, in so far as we know, there have been no comparative studies focusing on gender differences in medicine. This could be important because of the evident gap between men and women in the medical profession (34), although some studies have shown that gender stereotypes related to professionals in Spain have changed in recent times (35). Furthermore, type of contract is a variable of interest, since uncertain employment conditions could increase stress in the workplace, affecting emotional intelligence (31). However, and despite its importance, there is hardly anything in the literature about the relationship of sociodemographic and employment variables in emotional intelligence of medical professionals in active practice.

Self-esteem is how people value their skills and attributes as positive or negative, that is, the feelings that arise when they evaluate themselves (36). Self-esteem affects how they interpret and react to the challenges that arise in their surroundings (37). Therefore, low self-esteem can contribute to increasing job stress, and thereby, have repercussions on physicians' job performance and personal well-being. This could be a severe problem for society as a whole considering that some studies have shown that a fourth of physicians have low self-esteem, and workers with low self-esteem are almost three times as likely to suffer from stress and burnout (14).

Self-esteem is influenced by positive and negative feelings of professional self-concept, which are mainly generated by the perception of efficacy in clinical settings, job skills and professional assessment (27). It has been found that physicians' self-esteem increases with age (38), and that there are gender differences, in which men score higher in global self-esteem (39). As self-esteem is related to evaluation based on cultural norms, men and women evaluate their resources and specific abilities differently (40), and so during adulthood, in the medical profession women may be distanced from sociocultural expectations (34, 41), and this could diminish their self-esteem. Thus, the self-esteem of female physicians has been found to be lower than their male colleagues (39). Something similar occurs with age, where younger healthcare workers, by not meeting high social demands, show lower self-esteem, as mentioned in previous studies (14). For example, the study by Reyna-Figueroa et al. (42) mentioned that over 30% of medical residents had low self-esteem. Moreover, since self-esteem of employees is strongly conditioned by their job security and self-confidence (43), those who do not have a stable contract would probably have lower levels of self-esteem. It has also been found that the self-esteem of healthcare employees buffers their usually heavy workloads, helping them to stay on the job longer (44). Nevertheless, there is a gap in the literature on Spanish physicians in this regard which must be undertaken.

In addition, the emotional intelligence of healthcare professionals has been found to be positively related to self-esteem (45), and more specifically, Mood is a strong predictor of it (46). These results are similar to those found in samples of other workers (47) and groups (48). Therefore, as emotions are based on implicit beliefs and their experience, expression and management are driven by sociocultural values (49), it is of interest to know these constructs in the Spanish medical context. The objective of this study was to analyze the relationship between the two constructs (self-esteem and emotional intelligence) in physicians in active practice in Spain, and their relationship with sociodemographic variables (such as sex, age or number of children) and employment (such as type of contract).

Our hypothesis was that we would find the self-esteem of physicians to be positively related to emotional intelligence, especially mood (H1); with regard to sociodemographic variables, older physicians with children would have higher emotional intelligence and self-esteem (H2), men would have more capacity for adapting and higher self-esteem than their female counterparts; and lastly, related to job variables, employees with a permanent contract would have higher levels of self-esteem and emotional intelligence (H3).

## Materials and methods

### Study design and participants

The study had a descriptive, cross-sectional research design directed at Spanish physicians, following the STROBE Statement guidelines for reporting cross-sectional studies (50). Inclusion criteria were that they be actively employed at the time of the survey, and therefore, unemployed physicians were excluded. The original sample was made up of 192 physicians, of whom 12 were discarded (seven because they were unemployed at the time of data collection and five because they did not fill in the whole questionnaire). The final study sample consisted of a total of 180 physicians in active practice throughout Spain.

### Measurements

A questionnaire was prepared *ad hoc* for collecting participant sociodemographic data (age, sex, marital status, number of children) and employment situation/stability (permanent or temporary contract).

*Rosenberg Self-esteem Scale* (51). Developed for evaluating self-esteem, it is comprised of 10 items with content focused on feelings of respect and acceptance of oneself (e.g.: “In general, I feel satisfied with myself”). It is answered on a four-point Likert-type scale (1 = strongly agree, 2 = agree, 3 = disagree, 4 = strongly disagree). Its application in our study sample had a reliability of ω = 0.81, α = 0.82.

*Brief Emotional Intelligence Inventory for Senior Citizens* (52) validated and scaled for an adult Spanish population, adapted for an older population from the Emotional Intelligence Inventory: Young Version (EQ-i-YV) by Bar-On and Parker (53). It consists of 20 items with four answer choices on a Likert-type scale and is structured in five factors: Intrapersonal (e.g.: “I can describe my feelings easily”) (ω = 0.92, α = 0.91), Interpersonal (e.g.: “I understand well how other people feel”) (ω = 0.74, α = 0.72), Stress Management (e.g.: “When I get angry, I act without thinking”) (ω = 0.84, α = 0.83), Adaptability (e.g.: “I can solve problems different ways”) (ω = 0.74, α = 0.72), and Mood (e.g.: “I am happy with the kind of person I am”) (ω = 0.86, α = 0.85). Reliability of the complete scale was ω = 0.79, α = 0.85.

### Procedure and ethics approval

Participants were selected applying the inclusion and exclusion criteria described above. Before data were collected, participants were guaranteed compliance with information, confidentiality and ethical standards in data processing. Data were acquired using questionnaires implemented on an Internet platform that enabled participants to fill them in online, as a CAWI (Computer Assisted Web Interviewing) survey. The participants gave their informed consent by marking a box designated for the purpose, which then allowed them access to the questionnaire. They were also informed that they could stop answering and leave the study at any time without penalization of any kind.

### Data analysis

First, normality tests showed a non-normal distribution of data. Therefore, and in view of the sample size (N = 180) non-parametric statistics were used. To identify associations between the individual variables (emotional intelligence and global self-esteem), the Spearman's rho correlation coefficient was applied, and also the corresponding descriptive statistics. The between-group comparison results were also presented by sociodemographic characteristics and employment situation. Between-group differences were estimated for this using the Mann-Whitney U. In addition, the probability of superiority (PSest) was also calculated as the effect size of the Mann-Whitney U: this coefficient estimates the probability that a randomly selected score in one group will be higher than one selected from another group (54). The values proposed for interpreting the PSest as a measure of effect size are the following: ≤ 0 no effect, ≥ 0.56 small, ≥ 0.64 medium and ≥ 0.71 large (55).

Finally, to test for any differences in emotional intelligence between self-esteem groups (based on self-esteem scores proposed by Rosenberg: ≤ 25 low, 26–29 medium, and 30–40 high) (51). The Dunn (56, 57) test for nonparametric pairwise multiple comparisons in independent groups was used with the Holm (58) adjustment, which produces multiple comparisons following a Kruskal-Wallis k-way test (59).

Instrument reliability was examined by estimating the McDonald (60) Omega coefficient following the proposal and instructions of Ventura-León and Caycho (61). Statistical data treatment was done with SPSS v.24 (62) and jamovi v.1.8 (63).

## Results

### Sociodemographic descriptive statistics

The 180 physicians in active practice was aged 25 to 62 and mean age of 34.61 (SD = 6.69). By gender, 23.3% (n = 42) were men and 76.7% (n = 138) women, mean age 35.17 (SD = 6.06) and 34.44 (SD = 6.88) respectively. Their marital status was 55% (n = 99) married, 41.1% (n = 74) single, and the remaining 3.9% (n = 7) divorced or separated.

Finally, at the time of data collection, 53.9% (n = 97) had a temporary contract and 46.1% (n = 83) had a stable employment situation (Table 1).

**Table 1:** Sociodemographic characteristics of the sample.

**Characteristics**	**% (n)**	**M** ±**SD (age in years)**
Age		34.61 ± 6.69
Sex		
Male	23.3 (42)	35.17 ± 6.06
Female	76.7 (138)	34.44 ± 6.88
**Marital status**		
Married	55 (99)	
Single	41.1 (74)	
Divorced or separated	3.9 (7)	
Employment situation^*^		
Temporary (unstable)	53.9 (97)	
Permanent (stable)	46.1 (83)	

* Employment situation at the time of data collection. M, mean; SD, Standard Deviation.

### Emotional intelligence and self-esteem: Descriptive statistics and correlations

The correlation coefficients (Table 2) revealed that the emotional intelligence factors had positive correlations with Global Self-esteem in all cases. Medium relationships of Self-esteem with Stress management and Adaptability, and high with Mood were noteworthy.

**Table 2:** Emotional intelligence: descriptive statistics and correlation with global self-esteem (*N* = 180).

**Emotional intelligence**	**Mean**	**SD**	**Spearman's rho self-esteem**	**p-value**
Intrapersonal	10.100	2.744	0.167^*^	0.025
Interpersonal	11.676	1.668	0.136	0.068
Stress management	12.647	2.117	0.281^**^	<0.001
Adaptability	11.185	1.673	0.283^**^	<0.001
Mood	11.512	2.122	0.715^**^	<0.001

* p < 0.05;

** p < 0.01.

Concerning participant characteristics, the interpersonal factor of emotional intelligence was observed to have negative associations with age (r = −0.19, p < 0.05) and number of children (r = −0.237, p < 0.01). The rest of the components of emotional intelligence and self-esteem had no associations with age or number of children.

By participant gender (Table 3), statistically significant differences were observed with the emotional intelligence factors: Adaptability U = 2329 (Z = −1.988), p < 0.05, PSest = 0.40 (with a range mean in the groups of men and women of 104.51 and 86.24, respectively), and interpersonal U = 2309.5 (Z = −2.040), p < 0.05, PSest = 0.40 (with a range mean in the groups of men and women of 76.95 and 94.62, respectively).

**Table 3:** Emotional intelligence and self-esteem: comparison by gender.

**Variable**	**Gender**	**n**	**Range average**	**U**	**p**	**PS**_est_
Intrapersonal	Men	42	84.33	2639	0.373	0.45
	Women	138	92.38			
Interpersonal	Men	42	76.95	2329	0.047	0.40
	Women	138	94.62			
Stress management	Men	42	96.63	2640.5	0.374	0.45
	Women	138	88.63			
Adaptability	Men	42	104.51	2309.5	0.041	0.40
	Women	138	86.24			
Mood	Men	42	101.48	2437	0.110	0.42
	Women	138	87.16			
Global self-esteem	Men	42	102.50	2394	0.087	0.41
	Women	138	86.85			

U, U de Mann-Whitney; p, p-value, PS_est_, Probability of superiority (effect size).

Table 4 shows that there were no differences in statistical significance level in the Mann-Whitney U test results, although range averages with a probability of superiority near 0.50 were observed in all cases in the group of professionals with a stable employment situation compared to those with unstable employment.

**Table 4:** Group comparison by employment situation.

**Variable**	**Employment situation**	**n**	**Range average**	**U**	**p**	**PS**_est_
Intrapersonal	Unstable	97	88.65	3846.5	0.601	0.48
	Stable	83	92.66			
Interpersonal	Unstable	97	89.38	3917	0.48	0.49
	Stable	83	91.81			
Stress management	Unstable	97	87.97	3780.5	0.473	0.47
	Stable	83	93.45			
Adaptability	Unstable	97	88.90	3870	0.647	0.48
	Stable	83	92.37			
Mood	Unstable	97	86.17	3870	0.217	0.48
	Stable	83	95.56			
Global self-esteem	Unstable	97	87.05	3691	0.336	0.46
	Stable	83	94.53			

U, Mann-Whitney U; p, p-value; PS_est_, Probability of superiority (effect size).

### Emotional intelligence by physician self-esteem level

**Figure 1:**
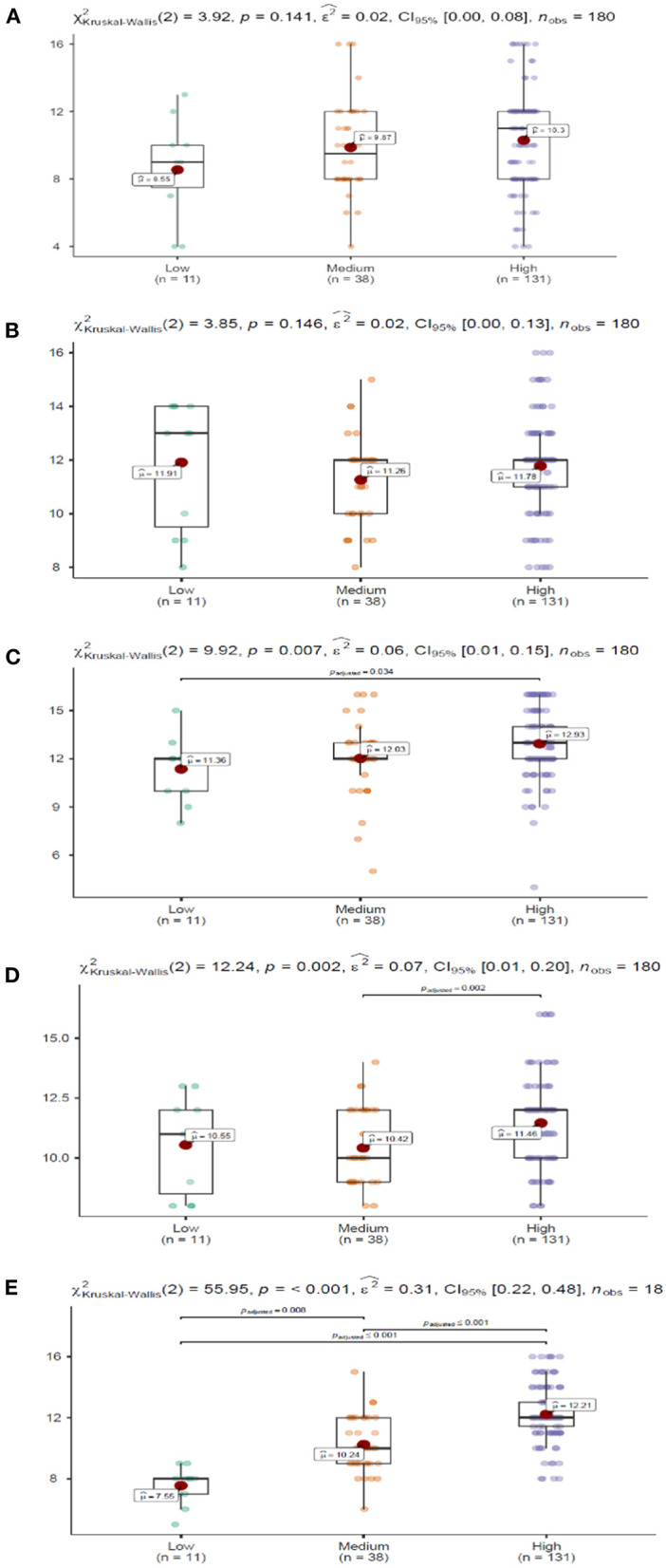
Emotional intelligence by self-esteem level. Dimensions: **(A)** Intrapersonal, **(B)** Interpersonal, **(C)** Stress Management, **(D)** Adaptability, and **(E)** Mood. Nonparametric pairwise multiple comparisons in independent groups using Dunn's test. Adjustment method (p-value): Holm.

The figures below show the results of the multiple comparison analysis for each dimension of emotional intelligence by level of self-esteem (Figure 1).

Significant differences were observed in Stress Management, specifically between the groups with high and low self-esteem (*p* < 0.05), where the high self-esteem group scored higher. In Adaptability, the differences were between professionals with medium and high self-esteem (p < 0.01), where those with high self-esteem scored significantly higher. And finally, Mood showed significant differences between the three self-esteem groups. Specifically, professionals with high self-esteem had the advantage over medium (p ≤ 0.001) and low (p ≤ 0.001) self-esteem. And in turn, those with medium levels of self-esteem had significantly higher levels (p < 0.01) in the Mood dimension compared to the low self-esteem group.

## Discussion

Because of the scarcity of studies related to emotions in the medical context, the objective of this study was to analyze the relationship between emotional intelligence and self-esteem, and also to determine the association of both constructs with sociodemographic and employment variables in a sample of Spanish physicians in active practice. The personal competencies of healthcare professionals are of growing importance, as the literature has demonstrated their relationship with job performance, job satisfaction and personal well-being (38), as well as leadership (11, 12). There is therefore a growing interest in knowing associated variables, such as emotional intelligence and self-esteem in physicians. This study is important because it is one of the first in describing how individual factors (such as gender or age) and employment (such as employment stability) are related to these variables in Spanish physicians.

In regard to the first objective posed, and according to our first hypothesis, global self-esteem of physicians was found to be associated positively with the Stress Management and Adaptability dimensions of emotional intelligence, and especially with Mood, with which it has a close relationship. These results are similar to those found in other groups (47, 48). Furthermore, studies such as the one by Pérez-Fuentes et al. (45) have shown that mood is an important predictor of self-esteem in healthcare workers. Regarding sociodemographic and employment factors, it was found that ability to understand and manage emotions diminished as the number of children and age increased. These striking results differ from what was originally expected and reject the second hypothesis posed. We speculate that this finding may be motivated by the fact that as physicians acquire more experience in dealing with their patients and their families grow, their skill in understanding and controlling the emotions of others is less than what they would expect, and thus this factor of emotional intelligence decreases. Nevertheless, new studies should analyze emotional intelligence as a function of age and children.

Moreover, on evaluating gender differences, it was found that men had better stress management and women had higher intrapersonal emotional intelligence. Thus, although these differences were not significant, the results of the effect size suggest that there are salient differences between groups (64). The results on the study's third hypothesis could be a reflection of the gender gap in the medical profession, as found by Pope (34) and others, that would cause men to feel more capable of facing changing and stressful situations. Therefore, gender has to be considered, especially with regard to program development, as mentioned for other healthcare workers (2). Especially considering that although gender stereotypes in the professions are changing (39), there is still a perceptible gap in the medical profession (34), which could diminish women's capacity for managing overwhelming emotions and adapting.

It was also found that the group of employees with a stable contract scored higher on all the emotional intelligence variables, with almost a small effect, partially confirming the study's fourth hypothesis.

This could be due to increased stress from the uncertainty of unstable employment conditions, as found by Giménez-Espert et al. (31). It should be mentioned that emotional intelligence evaluated with the EQ-I reports stable emotional intelligence dimensions (25) and higher predictive power (65). Also, based on the mixed model employed in this study, Emotional intelligence is understood to be a positive ability that can be trained. This emphasizes the possibility and need to improve emotional competence of physicians, especially women, who work with temporary contracts or have medium or low self-esteem (23). More so considering that EI es the most easily modifiable of the dispositional traits, so significant changes can be achieved with repercussions on life satisfaction of employees (7).

No relationships of self-esteem or the sociodemographic characteristics were found with any of the variables analyzed. These results are contrary to those found in samples of physicians in other countries, where it was found that self-esteem increased with age (38) and was higher in men (39). This could be due to the evaluation of self-concept in physicians in Spain being favorable and independent of gender or age. Since self-esteem is largely related to professional self-concept (27), it is not surprising that physicians have a high self-concept in view of the high sociocultural esteem of this profession. Emotional intelligence levels were also compared by their level of self-esteem. Physicians with high self-esteem showed better stress management and mood than those with low self-esteem. And with regard to the adaptability factor, physicians who had high global self-esteem score d higher in their ability to confront new situations and scenarios than professionals with medium levels of self-esteem. This stresses the importance of maintaining high levels of self-esteem in these professionals. Low self-esteem can cause psychological effects that make them more susceptible to stress. For example, the lack of assertiveness and passivity are two common effects of low self-esteem that cause employees to accept too many tasks or tasks that they cannot manage, which increases stress (14). In this sense, it has been shown that improving self-esteem can transform threatening changes into challenges (15). Therefore, based on the results found, and in view of the current need to train in personal competencies and skills necessary for proper professional performance (1, 10) and promotion of effective healthcare leaders (13), the relationship between global self-esteem and stress management, adaptability and mood factors of emotional intelligence is underlined. Professionals with high levels of self-esteem show higher levels in these determining factors of their professional performance. It is also important to deal with these variables, especially in women and those who do not have a permanent contract.

This study had some limitations. The young age of the participants limited findings related to this variable. The cross-sectional design of the study did not allow conclusions to be reached on the evolution of the variables analyzed, however, a longitudinal study could enable progress in the analysis of the variables included here. Finally, the area where the physicians worked was not taken into account, and due to the possible disparity of the adverse and stressful situations they could be subjected to depending on where they work, in future this should be a variable to consider.

## Conclusions

Since emotions are marked partly by cultural values, and as there are few studies on them in the field of medicine in Spain, we think that the results shown are valuable. This study contributes to understanding the relationships between the dimensions of emotional intelligence, self-esteem and personal and labor factors in medical professionals. Emotional intelligence has an important role for these employees whose work involves emotionally intense job characteristics or who must supervise others.

The findings demonstrate that there is a relationship between the levels of global self-esteem and capacity for regulating the overwhelming emotions, adaptability and positive mood in physicians. Healthcare management and administration should consider self-esteem and emotional intelligence measures, especially of women and those who have temporary contracts, and offer employees prevention programs and intervention training in these personal competencies. In clinical practice, improving physician's conditions and personal resources, such as self-esteem and emotional intelligence could have repercussions on their job performance, increasing leadership, and in fact, making a significant difference in medical attention.

## Data availability statement

The data presented in the current study are available from the corresponding author on reasonable request.

## Ethics statement

The studies involving human participants were reviewed and approved by University of Almería Bioethics Committee (Ref: UALBIO2019/031). The patients/participants provided their written informed consent to participate in this study.

## Author contributions

MP-F and MM contributed to the concept, design, analysis, and interpretation of the data. ÁM contributed to the technical details and manuscript preparation. MP-F, MM, and ÁM wrote the manuscript. JG contributed to critically revising the manuscript for important intellectual content and the final approval of the version to be published. MM, MP-F, ÁM, and JG accepted, agreed that the work is original any methods and data presented are described accurately, honestly, and any relevant interests have been disclosed. All authors contributed to the article and approved the submitted version

## Conflict of interest

The authors declare that the research was conducted in the absence of any commercial or financial relationships that could be construed as a potential conflict of interest.

## Publisher's note

All claims expressed in this article are solely those of the authors and do not necessarily represent those of their affiliated organizations, or those of the publisher, the editors and the reviewers. Any product that may be evaluated in this article, or claim that may be made by its manufacturer, is not guaranteed or endorsed by the publisher.
